# Salivation by Chemical Action in the Stomach

**Published:** 1847-12

**Authors:** J. S. Jones

**Affiliations:** Covington, Ind.


### ARTICLE IV.

*Salivation by Chemical action in the Stomach.* By J. S. Jones, M. D., of Covington, Ind.

Braithwait, in his *Retrospect*, No. I, Part 1st, page 45, notices, what the *London Lancet* calls an ingenious explanation, by Mr. Snow, in a discussion in the Westminster Medical Society, of the occurrence of salivation by mercury more readily at one time than at another. As will be recollected, he supposed it to be "the result of the presence of acid in the first passages, converting the blue mass, or the calomel, into corrosive sublimate." He came to the conclusion, by often seeing patients salivated by taking a small portion, at the same time with mixtures containing sulphuric acid.

My intention is only to inquire, whether Mr. Snow's position merits thought; and if it does—whether sulph. acid is indispensible in the chemical process?

Although the British Colleges adhere to the old nomenclature, muriate and sub. mur., since not only our best chemists, but the decision of the U. S. Convention in the revision of our Pharmacopœia, in 1830, justifies our calling them the proto and bi chlorides, we will use these terms.

It is well known that chlorine and mercury, at common temperatures, combine with one equivalent of each, producing the proto-chloride, or calomel, but when heated they combine two to one, forming the bi-chloride (Beck's Chem., page 365). Knowing full well that all the juices of the stomach are saturated with chloride of sodium, the question arises, is the heat of the stomach sufficient to cause a union of two parts of chlorine to one part of mercury? If so, we must conclude that mercury acts only as a bi-chloride, and that unpleasant effects depend on the speedy chemical union—that when it is slow, injury is not as likely to be produced.

Sulphuric acid may facilitate the operation, by combining with the soda, forming sulph. soda, and leaving the chlorine free for a more ready combination with the mercury; but without the help of this acid, we are aware that muriate of sodium, mercury, and caloric, will generate corrosive sublimate—then why not in the stomach as well as in the crucible. But what is it that facilitates or retards the chemical process, at one time affording enough to produce all the unhappy effects, and at another time scarcely being perceptible, regardless, too, of the use of sulphuric acid mixtures, as observation shows. Does it depend on the quantity of muriatic acid in the stomach at the time, or can there be a better cause assigned?

Mr. Snow considered the remedy to be in the use of the bi-chloride at once, and in suitable quantities.

If blue mass or calomel is preferable, can they not be combined with the proto-chloride of tin; chlorine having a stronger affinity for tin than mercury, bi-chloride of tin will be the first production, using up the chlorine, and thus preventing a combination with the mercury. The points in question, are:

1st. Can chloride of sodium and mercury remain together in the stomach, without producing the chloride of mercury?

2nd. Is the heat of the stomach sufficient to produce the bi-chloride?

3d. What facilitates or retards the process?

4th. What would be the effect of proto-chloride of tin used in connection with mercury?

If the subject merits attention, we hope to hear from the profession respecting it.

—

Our leisure has not been sufficient to permit us to examine the subject as we would like, before attempting to answer the queries of our correspondent. We accordingly lay his article before our readers, that they may acquaint us with any facts in their possession, which may bear upon the subject, and hope that we may, by our next number, find time to institute some experiments which may perhaps enlighten us on the points proposed. J. V. Z. B.

